# Persistent Motion Sensation: A Case Report of Mal De Débarquement Syndrome in a Long-Term Boat Dweller

**DOI:** 10.7759/cureus.79342

**Published:** 2025-02-20

**Authors:** Bola Habeb, Asaad Mouselli, Otto Valdes, Seth Fowler

**Affiliations:** 1 Internal Medicine, University of Florida College of Medicine / Ascension Sacred Heart, Pensacola, USA

**Keywords:** cruise ship syndrome, maladaptive neural plasticity, mal de débarquement syndrome, motion-triggered dizziness, post-motion syndrome, sensory integration, vestibular dysfunction, vestibular rehabilitation therapy

## Abstract

Mal de débarquement syndrome (MdDS) is a rare neurological condition characterized by a persistent sensation of motion, such as rocking, swaying, or bobbing, typically arising after exposure to passive motion, such as during a cruise, flight, or car travel. Unlike motion sickness, MdDS symptoms often begin after the motion ceases and can persist for weeks, months, or even years, significantly impacting the quality of life. Although the exact pathophysiology remains unclear, it is thought to involve maladaptive neural plasticity in the vestibular and sensory integration systems. Diagnosis is primarily clinical, requiring careful exclusion of other vestibular and neurological disorders. Treatment approaches, including vestibular rehabilitation therapy, transcranial magnetic stimulation, and symptom management, show variable success. This article summarizes the current understanding of MdDS, its clinical features, diagnostic criteria, and therapeutic strategies, emphasizing the need for increased awareness and further research into this debilitating condition.

## Introduction

Mal de débarquement syndrome (MdDS), first described in the late 19th century, is a poorly understood neurological condition characterized by a persistent illusion of self-motion, often described as a rocking or swaying sensation [1]. Unlike motion sickness, MdDS typically manifests after the cessation of motion, such as following a cruise, flight, or long car journey. While transient symptoms are common and resolve within hours to days for most individuals, MdDS is diagnosed when symptoms persist for at least one month and cannot be attributed to other vestibular or neurological disorders [2].

The pathophysiology of MdDS remains unclear, although it is hypothesized to involve maladaptation of the brain's vestibular and somatosensory systems to prolonged motion stimuli. Risk factors include female sex, middle age, and prolonged exposure to passive motion [1]. The condition can significantly impair daily functioning, leading to physical, emotional, and social challenges for affected individuals.

The International Classification of Vestibular Disorders defines vertigo as the “sensation of self-motion when no self-motion is occurring or the sensation of distorted self-motion during an otherwise normal head movement.” Within this broad definition, the experience of MdDS can be categorized as a form of vertigo. However, unlike the intuitive perception of vertigo as a rotational sensation, MdDS presents as an oscillatory or periodic sensation. This motion is perceived even at rest, without any head movement, and is typically described as a rocking, bobbing, or swaying sensation rather than spinning [3].

This case report discusses the clinical presentation, diagnostic approach, and management of a patient with MdDS, providing insights into the condition's impact and potential therapeutic strategies. Through this report, we aim to raise awareness about MdDS among physicians to facilitate early diagnosis, reducing the need for multiple consultations, repeated imaging studies, and unnecessary healthcare expenses.

## Case presentation

A 71-year-old male with a medical history of hypertension, hyperlipidemia, benign paroxysmal positional vertigo (BPPV), depression, anxiety, and post-traumatic stress disorder (PTSD) presented to our facility for evaluation following a ground-level fall accompanied by loss of consciousness. The patient reported a several-week history of progressive dizziness, unsteadiness, and episodes of dissociation, prompting further investigation.

The patient has lived on a boat docked in a harbor for the last 10 years. He described a persistent sensation of "walking on water" and feeling unsteady on his feet, particularly in the morning upon disembarking from the boat. These symptoms were refractory to his usual Dix-Hallpike maneuvers, which he performed independently for his known BPPV. He also reported multiple ground-level falls, with his most recent fall resulting in a brief loss of consciousness. Admission laboratory studies were unremarkable (Table 1).

**Table 1:** Admission laboratory data. BUN: blood urea nitrogen; AST: aspartate aminotransferase; ALT: alanine aminotransferase; TSH: thyroid-stimulating hormone

Parameters	Reference range, adults	Patient's values on admission
Hemoglobin (g/dL)	12.0–15.5	12.8
Hematocrit (%)	34.9–44.5	37.7
White cell count (per mm3)	3500–10500	7300
Platelet count (per mm^3^)	150,000–450,000	245,000
Sodium (mEq/dL)	135–145	140
Potassium (mEq/dL)	3.5–5.1	4.4
Bicarbonate (mEq/dL)	22–29	24
BUN (mg/dL)	12–21	12
Creatinine (mg/dL)	0.7–1.2	0.8
Magnesium (mg/dL)	1.6–2.6	1.9
Phosphorus (mg/dL)	2.3–4.7	2.9
AST (units/L)	12–31	22
ALT (units/L)	9–29	30
Total bilirubin (mg/dL)	0.1–1.1	1.1
TSH (mcIU/mL)	0.35–3.9	1.7
Vitamin B12 (pg/mL)	213-816	420
Folate (ng/mL)	7-31.4	11.2
Ethanol (mg/dL)	<10	<10

**Figure 1:**
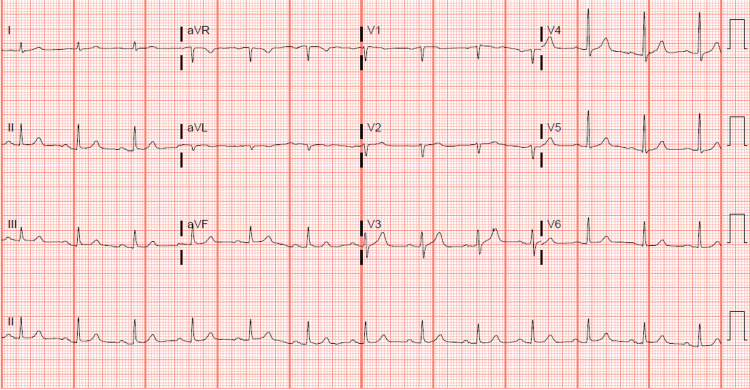
12-lead EKG showing normal sinus rhythm. EKG: electrocardiogram

**Figure 2:**
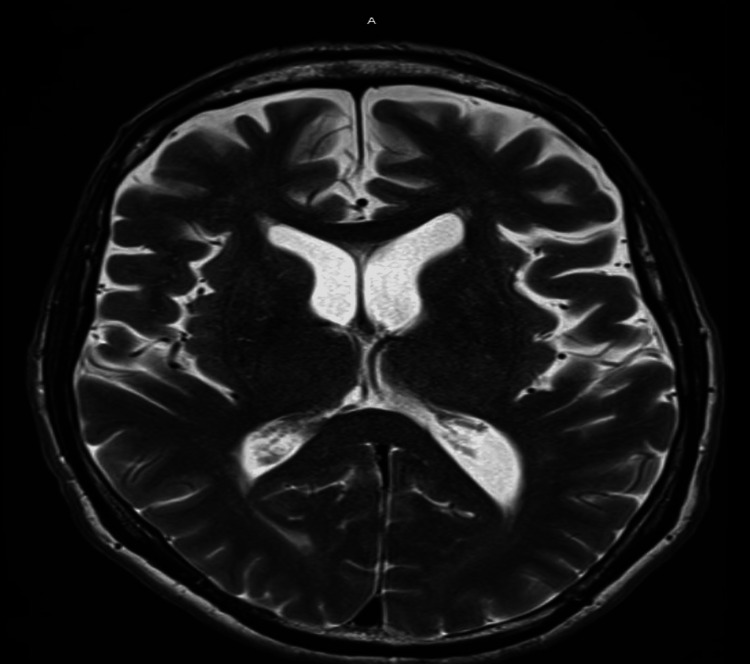
MRI brain showing normal findings. MRI: magnetic resonance imaging

A comprehensive physical examination, along with a thorough neurological evaluation, revealed no focal deficits or abnormalities. An extensive diagnostic workup, including an electrocardiogram (EKG), computed tomography (CT) of the brain, computed tomography angiography (CTA) of the head and neck, and magnetic resonance imaging (MRI) of the brain, showed no signs of acute pathology or structural intracranial abnormalities, such as stroke, carotid dissection, or acoustic neuroma (Figures 1, 2).

The patient had previously been evaluated by an ENT specialist for similar symptoms, with video-nystagmography (VNG), oculomotor testing, and audiogram all returning normal results.

Given the patient’s history of prolonged exposure to passive motion (living on a boat), the characteristic sensation of rocking or swaying, and the temporal pattern of symptom exacerbation in the morning, a diagnosis of MdDS was made. This diagnosis was further supported by the exclusion of other neurological, vestibular, and structural causes through extensive testing.

The patient was prescribed vilazodone, a benzodiazepine, to address his symptoms of dizziness, anxiety, and depression. He was advised to discontinue living on the boat to minimize exposure to passive motion, a known trigger for MdDS. The patient was reassured that his symptoms would likely resolve spontaneously over time. He was closely monitored in the neurology outpatient clinic, with gradual improvement noted over the following months.

## Discussion

MdDS is a rare and often underdiagnosed neurological condition characterized by a persistent sensation of motion, such as rocking, swaying, or bobbing, typically arising after exposure to passive motion [2]. While transient motion-related sensations are common and self-limiting, MdDS is distinguished by its chronicity, lasting weeks, months, or even years, and its significant impact on quality of life. It is widely recognized as a rare neurological disorder, with a reported prevalence of 1.3% among patients attending a neuro-otological clinic [4].

The exact mechanisms underlying MdDS remain poorly understood. MdDS is a complex disorder with several hypothesized pathophysiological mechanisms. One key hypothesis suggests a malfunction in the brain's velocity storage mechanism, leading to a persistent "motion memory loop" after prolonged motion exposure [5]. Another theory proposes that sensory mismatch between vestibular, visual, and proprioceptive inputs causes maladaptive neural plasticity, resulting in perceived motion despite stability [6]. Dysfunction in cerebellar and cortical regions involved in motion processing has also been implicated, supported by abnormal activity observed in functional imaging studies [7]. In addition, hormonal fluctuations (e.g., estrogen levels) and genetic factors may influence MdDS onset and severity, particularly in women [8]. These hypotheses highlight the multifactorial nature of MdDS, emphasizing the need for further research to fully understand and treat this condition.

Patients with MdDS commonly describe their symptoms as a continuous sensation of rocking, swaying, or bobbing, often alleviated by re-exposure to passive motion, such as being in a car. Unlike traditional vertigo, MdDS does not involve spinning sensations [3]. Symptoms are typically exacerbated by visual stimuli, fatigue, or stress, further complicating daily activities and reducing the patient’s quality of life.

MdDS is a diagnosis of exclusion, requiring the careful elimination of other vestibular, neurological, and systemic conditions. Comprehensive history-taking is crucial, focusing on the temporal relationship between symptom onset and exposure to passive motion. Diagnostic tools such as vestibular function tests and imaging studies are often normal, underscoring the importance of clinical expertise in recognizing the condition.

Management of MdDS remains challenging due to the lack of standardized treatment protocols. Vestibular rehabilitation therapy (VRT) is commonly employed, focusing on habituation exercises to recalibrate the vestibular system. Emerging treatments, such as repetitive transcranial magnetic stimulation (rTMS), have shown promise in modulating neural activity in affected regions. Pharmacological interventions, including benzodiazepines and antidepressants, may provide symptomatic relief for some patients [2].

The rarity and limited awareness of MdDS often cause delays in diagnosis, leading to unnecessary tests and prolonged distress for patients. MdDS greatly impacts quality of life and imposes a substantial economic burden on those affected [9]. Increased recognition of the condition among healthcare providers is essential to improve diagnostic accuracy and expedite treatment. In addition, further research into the pathophysiology of MdDS is needed to develop targeted therapies. Collaborative efforts between neurologists, otolaryngologists, and rehabilitation specialists will be crucial in advancing the understanding and management of this debilitating condition.

## Conclusions

MdDS is a rare neurological condition that can severely disrupt a person’s quality of life. Despite its significant impact, MdDS remains largely underrecognized, leading to diagnostic delays and unnecessary tests. This lack of awareness, combined with the difficulty in diagnosing the condition, contributes to prolonged distress for patients who may struggle to find effective treatment options. As a result, MdDS not only affects the physical and emotional well-being of individuals but also places a considerable burden on healthcare systems.

The path to better management and treatment of MdDS requires greater awareness among both the public and healthcare professionals. Timely diagnosis and targeted interventions can help reduce the emotional and economic toll on those affected by the condition. With more research and improved recognition, it is possible to develop more effective therapies and support systems, ultimately enhancing the lives of those dealing with this debilitating disorder. Raising awareness about MdDS is essential to ensure that patients receive the care they need and are not left to suffer in silence.
